# Early repair of necrotic lesion of the femoral head after high-degree posterior rotational osteotomy in young patients—a study evaluated by volume measurement using magnetic resonance imaging

**DOI:** 10.1093/jhps/hnv021

**Published:** 2015-03-30

**Authors:** Tsubasa Ishikwa, Takashi Atsumi, Satoshi Tamaoki, Ryosuke Nakanishi, Minoru Watanabe, Yasuoki Kobayashi, Satoe Tanabe, Toshihisa Kajiwara

**Affiliations:** Department of Orthopaedic Surgery, Fujigaoka Hospital, Showa University School of Medicine, Yokohama, Japan

## Abstract

We investigated the repair of femoral head necrosis with extensive necrotic lesions treated by high-degree posterior rotational osteotomy (HDPRO) in young adults and adolescents (mean age; 30.8 years) using magnetic resonance imaging (MRI). HDPRO was performed on 72 hips from 66 cases, and of those, 60 hips from 60 cases were included in this study for data analysis. All cases had extensive collapsed lesion preoperative anteroposterior radiographs. In total, 34 hips were male and 26 were females. In total, 19 had a history of steroid administration, 11 had a previous femoral neck fracture, 7 had no particular etiologic factor, and 4 had a followed slipped capital femoral epiphysis. Antero-inferior viable areas were transferred to the loaded portion below the acetabular roof by this operation. The mean posterior rotational angle was 118.5°. MRI was taken after 1 month, 6 months and 1-year post-operatively. Post-operative necrotic lesion volume compared with the preoperative necrotic lesion volume was defined as lesion volume ratio (%). The reduction of necrotic volume was observed over time, and at 1 year post-operatively, it was 19.4% for patients in their teens, 35.3% for those in twenties, 42.8% for those in their thirties and 59.5% for those in their forties. From this study, we concluded that the extensive necrotic lesions decreased in size within a short period after HDPRO in young patients.

## INTRODUCTION

Femoral head osteonecrosis is a disease that develops in relation to high dose steroid therapy, heavy intake of alcohol and femoral neck fracture, etc. Many cases occur in patients under the age of 50, and when the necrotic lesion is extensive, the disease becomes progressive and intractable with bony collapse occurring early, and causes dysfunction of the hip joint as a weight-bearing joint [1].

 Hip arthroplasty performed for highly active young patients does not lead to problems in the short and mid-term, but in the long-term, the possibility of revision arthroplasty becomes a concern [2, 3]. Resurfacing for the purpose of bone conservation is often performed, but its long-term results are unknown [4]. Therefore, joint preservation treatment is the desired treatment option for younger patients. Core decompression is reported to be effective for early lesions [5], but nothing is stated regarding the size of the necrotic lesion on the acetabular weight-bearing area that is involved in the progression of the collapse. The long-term results of vascularized fibular grafting are favorable if the collapse is mild but the extent of preoperative necrosis is not reported [6]. Regarding vascularized iliac bone grafting, it has been reported that good results can be expected if the preoperative collapse is mild, but it has not been indicated if collapse already exists preoperatively or if the necrotic lesion is extensive [7]. Curved intertrochanteric varus osteotomy is an extra-articular osteotomy that reduces the dissection of muscles and does not cut the capsular ligament [9]. This less invasive procedure when compared with hip arthroplasty and femoral rotational osteotomy renders favorable results but can only be indicated in cases with a viable region on the lateral side of the femoral head [8, 9]. Good results for anterior rotational osteotomy are reported for early lesions without collapse of the head [10, 11]. However, in cases with advanced collapse of the head, which was present prior to surgery after anterior rotation, the collapsed necrotic lesion was located on the anterior side of the joint, leading to joint instability during motion [12]. Thus, there is a possibility of post-operative progression of arthropathic change that follows; thus, in cases with advanced collapse of the head, results are poor [11, 13].

Atsumi *et al*. [14–19] have performed high-degree posterior rotational osteotomy (HDPRO) for young patients who were not indicated for anterior rotational osteotomy or varus osteotomy because of their extensive necrotic areas. The concept of HDPRO is that after posterior rotation, the posterior column artery moves medially and is relaxed; therefore, tension does not occur, and because the vascular supply of the head can easily be preserved, HDPRO becomes possible [15]. In addition, after posterior rotation, a large spherical viable region will be located between the center of the head and its anterolateral side, so when the patient moves into a flexed position, which often occurs in one’s daily life, the better viable region would move towards the acetabular weight-bearing area and would bear the weight, allowing the head to constantly remain mechanically stable within the acetabulum [16]. Atsumi *et al*. [14–19] reported good results even in cases with large necrosis and prominent collapse, which ware not indicated for anterior rotational osteotomy or varus osteotomy.

Magnetic resonance imaging (MRI) for femoral head osteonecrosis enables the evaluation of necrotic lesions that cannot be detected by plain X-rays, and is a useful imaging tool [20]. A band-like low intensity area on the T1-weighted image that differentiates the necrotic lesion from the viable region shows histological cellular restorative reaction, highly vascular granular tissues, and fibrotic restorative reactions [21]. However, in cases with bone marrow edema or fibrotic invasion, the border becomes unclear [22]. In contrast, in fat suppression images, repair reaction generated at the border of the necrotic and viable regions could be depicted with good contrast [23].

Atsumi *et al*. [16, 17] reported that HDPRO is an effective joint preservation surgery by showing that after performing HDPRO to large femoral head osteonecrosis, it was confirmed by plain X-ray that post-operative necrotic lesion remodeling had taken place, subchondoral necrotic trabecular fracture had disappeared, and that the collapsed necrotic lesion that had migrated medially had regained its spherical contour.

The objective of this study was to evaluate the effectiveness of HDPRO for repairing collapsed necrotic femoral head that was moved medially and to investigate the necrotic volume of femoral head with post-operative MRI.

## MATERIAL AND METHODS

HDPRO (all cases were operated by one of the authors; T.A.) was performed on 72 joints for traumatic and non-traumatic femoral head osteonecrosis in 66 young patients (all below the age of 50) with extensive necrosis, between August 2003 and May 2012. Out of these cases, 60 joints from 60 cases were selected as study subjects. They were free from the progressive collapse and joint space narrowing as well as the ones in whom post-operative MRI was possible ∼1 month (26–39 days), 6 months (160–210 days) and 1 year (340–383 days) after surgery. In total, 12 joints were excluded from the study for the following reasons: 10 joints were excluded because the necrotic lesion was difficult to measure because of metallic artefacts; one joint was excluded on the basis of preoperative imaging that resulted in an early collapse; and one case was excluded from the study because of a post-operative deep infection.

From a total of 60 joints of 60 cases, 34 were males and 26 were females, and the mean age at the time of surgery was 30.8 years (12–49 years). In terms of the etiologic factors of femoral head osteonecrosis, in 45 hips of non-traumatic cases, 19 cases had a history of heavy alcohol intake, 19 cases had a history of high-dose steroid administration and 7 cases had without no particular etiologic factor. Underlying diseases for steroid administration were systemic lupus erythematosus in 7 cases, glomerulonephritis in 4 cases, mixed connective tissue disease in 3 cases, lymphatic leukemia in 2 cases, interstitial pneumonia in one case, malignant lymphoma in one case and facial nerve palsy in one case. In 15 hips of traumatic necrotic cases, there were 11 cases of necrosis after femoral neck fracture and 4 cases of necrosis following slipped capital femoral epiphysis (the necrosis following slipped capital femoral epiphysis was included in traumatic necrosis).

**Fig. 1.**
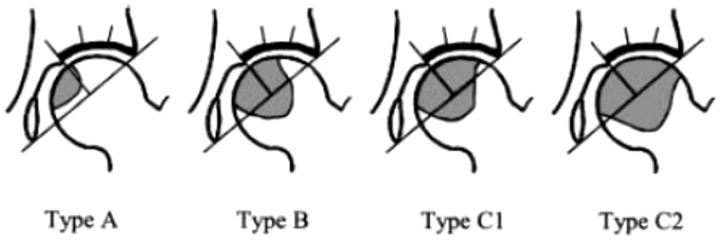
2001 revised classification of osteonecrosis.

With regard to the extent of preoperative necrotic lesion, the viable region occupied one-third or less of the lateral edge of the acetabular weight-bearing area (Type C1) on plain X-ray AP images in 11 joints out of 60. No viable regions were observed on the acetabular weight-bearing areas on plain AP X-ray images (Type C2) in 49 joints (for these types, the criteria of the Japanese Investigations Committee was applied) [24] (Fig. 1). In all 60 joints, head collapse was observed. On plain AP X-ray images, in 29 joints, head collapse was <3 mm and joint space narrowing was not observed (Stage 3A). In 25 joints, head collapse was 3 mm or more and without joint space narrowing (Stage 3B). In 6 joints, joint space narrowing was observed (Stage 4) that was also associated with 3 mm or more head collapse. (for these stages, the criteria of the Japanese Investigations Committee was applied) [24]. In 24 cases out of 60, femoral head osteonecrosis on the contralateral side was also observed on plain X-ray of MRI. Out of these, two cases underwent hip joint replacement, two cases underwent varus osteotomy, three cases had posterior rotational osteotomy, and one case received anterior rotational osteotomy. Sixteen cases were asymptomatic and were left untreated. All of the contralateral sides were excluded from the study.

The angle of posterior rotation given had a mean of 118.5° (110–135°) and the added varus angle had mean of 20° (15–25°). In all 60 joints of 60 cases, bone scintigraphy was performed 6 weeks post-operatively that suggested that the vascular supply of the head was maintained in all cases.

For post-operative treatment, the use of a wheel-chair 2 weeks after surgery was allowed. From 5 to 6 weeks post-operatively, partial weight-bearing using two crutches was started and until 6 months post-operatively, walking with one crutch was mandated.

After HDPRO, all 60 cases of femoral head collapse on major loaded portion below the acetabulum did not occur in the last follow-up with plain AP radiograph and MRI, because uncollapsed anterior viable areas were transferred to the loaded potion below the acetabular roof and the collapsed necrotic focus was moved to the medial portion of the femoral head.

### Measurement by MRI

**Fig. 2.**
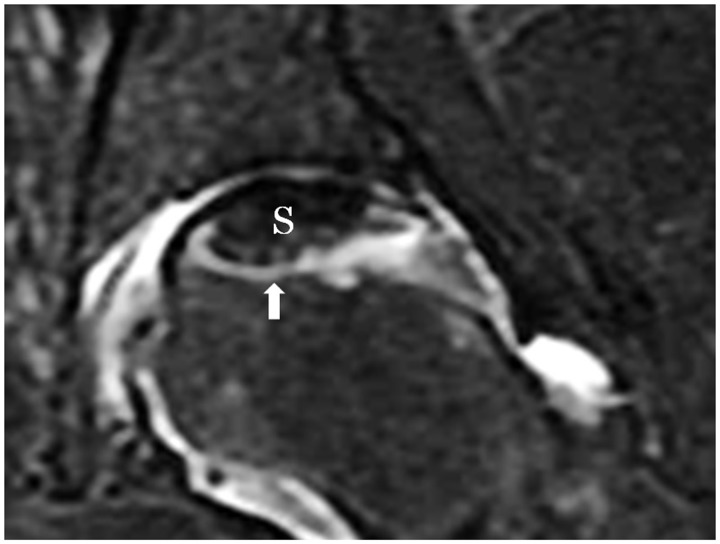
MRI T2-weighted fat suppression coronal image.

To quantitatively evaluate the necrotic lesion, necrotic lesion volume was measured by MRI. For MRI, the superconductive MR device MAGNETOM Vision (NUMARIS Version: VB3D manufactured by SIEMENS: static magnetic field strength 1.5 T) was used and images were taken with 5-mm intervals and 0.5-mm gaps. To judge the extent of necrotic lesions, band-like high-intensity area observed on the T2-weighted fat suppression coronal images showed restorative reaction [21], and it served as the border between the necrotic lesion and the viable region so the low-intensity area located proximally from the band-like high intensity area was judged as the necrotic area (S) (see Fig. 2). Using image measurement software (Pixs 2000-Pro; manufactured by Inotech), necrotic areas (S mm^2^) were measured. The low-intensity areas on each slice (Sn mm^2^) were measured and added as total volume (Vn mm^3^). The necrotic lesion volume was expressed by the following formula:

$${\text{Vn mm}}^{3}=\sum \left\{\text{Sn}\times 5.5\text{mm}\right\}\left(\text{5}.0\text{thk}/0.5\text{sp}\right).$$

The necrotic lesion volume ratio (%) was defined as the ratio between the preoperative necrotic lesion volume and post-operative necrotic lesion volume. The necrotic lesion volume ratio was expressed by the following formula:

$$\begin{array}{l} \text{Necrotic lesion volume ratio }\left(\%\right)\\ =\text{Postoperative Vn }\left({\text{mm}}^{\text{3}}\right)/\text{Preoperative Vn }\left({\text{mm}}^{\text{3}}\right)\;\times \text{1}00. \end{array}$$

The same investigator (T.I.) measured the necrotic lesion volume three times using the image analysis software, and generated the necrotic lesion volume ratio (%) in the form of mean values (range).

#### Method 1

Sixty joints for which HDPRO was performed and MRIs were taken ∼1 month, 6 months and 1 year after surgery, and were classified according to age groups: 15 joints from the group in their teens (mean age; 15.8 years), 12 joints from the group in their twenties (mean age; 25.5 years), 19 joints from the group in their thirties (mean age; 35.5 years) and 14 joints from the group in their forties (mean age; 45.2 years), and the necrotic lesion volume ratio (%) was measured to conduct a comparison with time.

#### Method 2

Sixty joints for which HDPRO was performed and MRIs were taken ∼1 month, 6 months and 1 year after surgery, were classified into two groups: one with 15 cases of traumatic femoral head necrosis (mean age; 29.3 years) and the other with 45 cases of non-traumatic femoral head necrosis (mean age; 31.3 years) to make a chronological comparison by measuring the post-operative necrotic lesion volume ratio (%).

#### Method 3

Sixty joints for which the MRIs were taken were classified into the following groups: 29 joints (mean age; 31.1 years) with a preoperative head collapse of <3 mm (Stage 3A), 25 joints (mean age; 30.8 years) with a preoperative head collapse of 3 mm or more (Stage 3B), and 6 joints (mean age; 29.3 years) with joint space narrowing (Stage 4) and 3 mm or more head collapse. Post-operative necrotic lesion volume ratios (%) were measured and chronological comparisons were made. For the statistical analysis of the inter-group comparison, Kruskal -Wallis H-test and Mann -Whitney U test was used for testing the significance level. For all tests, *P* < 0.05 was considered significant. JMP Pro 11.0.0 was used for statistical analysis.

## RESULTS

### Necrotic lesion volume ratio according to age

**Fig. 3.**
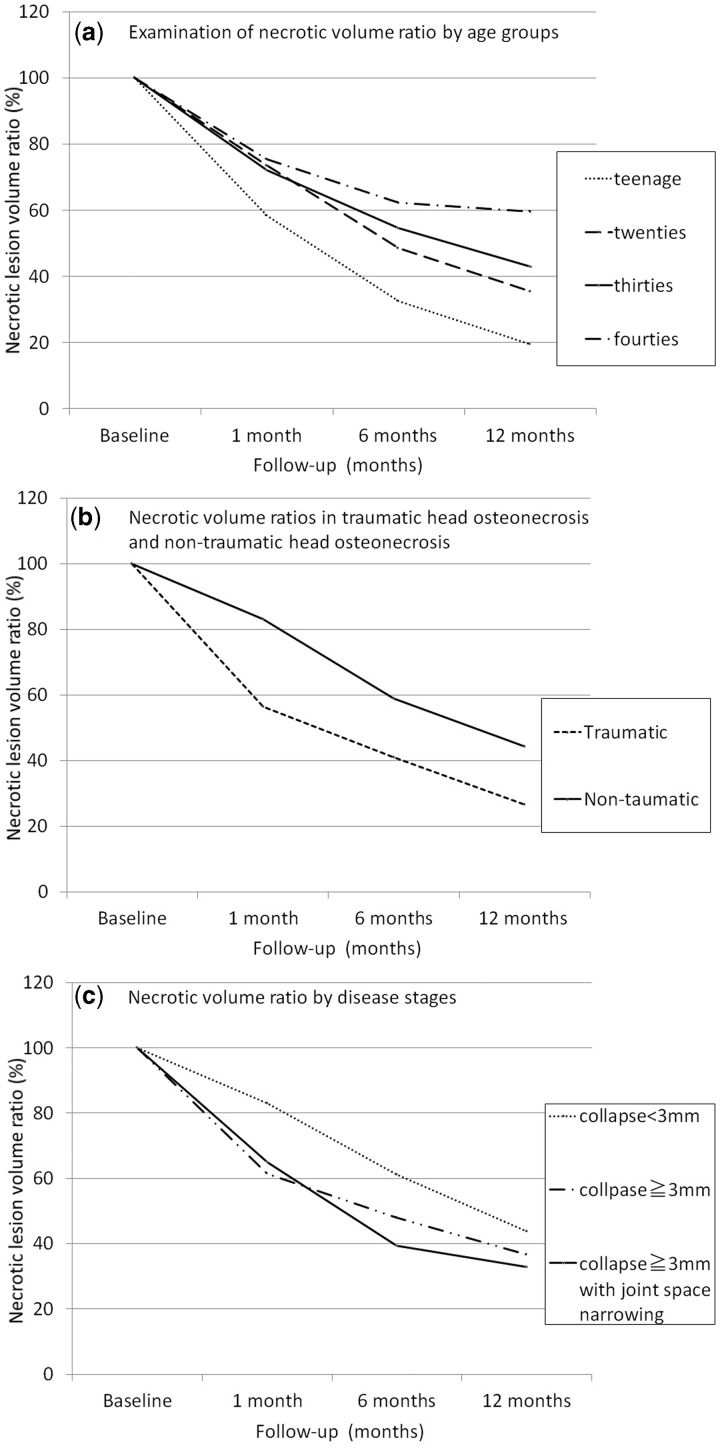
**(a)** Necrotic volume ratio by age groups. **(b)** Necrotic volume ratio in traumatic head osteonecrosis and non-traumatic head osteonecrosis. **(c)** Necrotic volume ratio by each stages.

The necrotic volume ratio (%) on the basis of age that was estimated after 1-year post-operatively was 19.4% (range; 6.5–28.2%) for the group in their teens, 35.3% (range; 10.5–43%) for the group in their twenties, 42.8% (range; 25.2–64.3%) for the group in their thirties and 59.5% (range; 35.3–96.1%) for the group in their forties, and for each age group, a reduction of necrotic volume was observed with time. In particular, in the teenager group, the repair of the post-operative necrotic region tended to be favorable compared with that of the patients in their twenties (*P* = 0.03, Mann-Whitney U test) (Fig. 3a).

**Fig. 4.**
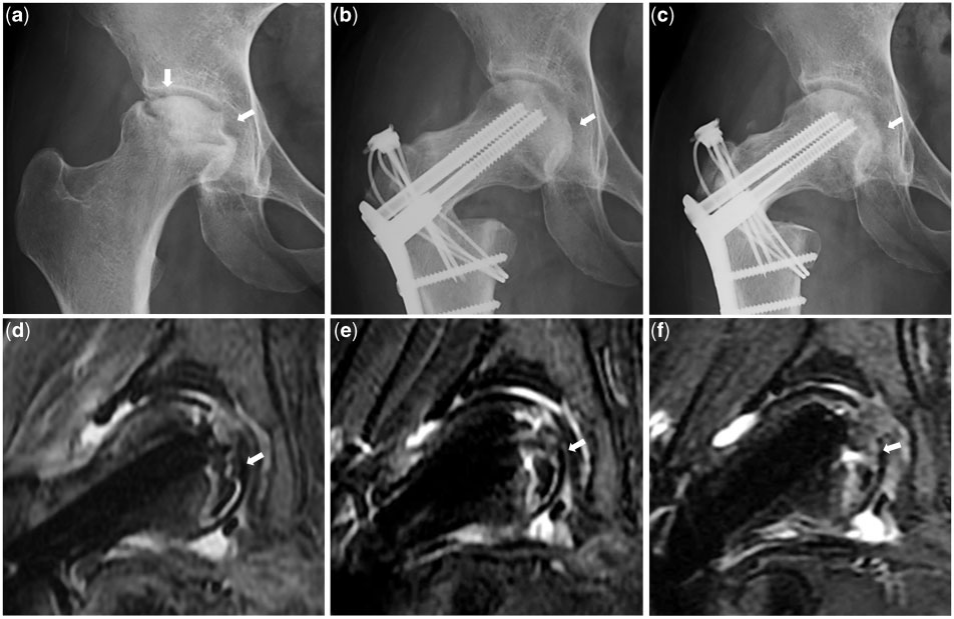
**(a)** Preoperative plain AP X-ray. **(b)** AP X-ray 1 month after operation. **(c)** One year after operation. **(d)** MRI T2 fat suppression coronary image 1 month after operation. **(e)** 6 months after operation. **(f)** One year after operation.

### Necrotic lesion volume ratio in traumatic head osteonecrosis and non-traumatic head osteonecrosis

When the necrotic lesion volume ratio was compared between traumatic head osteonecrosis and non-traumatic head osteonecrosis, 1 year after surgery, it was 26.6% (6.5–59.1%) in 15 cases of traumatic head osteonecrosis and 44.4% (22.4–96.1%) in 45 cases of non-traumatic head osteonecrosis, so good results for the repair of necrotic lesions were achieved in traumatic head osteonecrosis (*P* = 0.02, Mann- Whitney *U* test) (Fig. 3b).

### Examination of necrotic lesion volume ratio according to the disease stages

On comparing the necrotic lesion volume ratio according to the disease stages 1 year after surgery, it was found to be as follows; 43.7% (6.5–78.3%) for 29 joints for which preoperative head collapse was <3 mm, 36.6% (10.2–56.3%) for 25 joints for which preoperative head collapse was 3 mm or more and 32.9% (8.8–96.1%) for 6 joints for which preoperative head collapse was 3 mm or more accompanied with joint space narrowing. At 1 year after surgery, there were no significant differences in terms of necrotic lesion repair between cases with 3 mm or more progression of preoperative head collapse and those with <3 mm progression before surgery (*P* = 0.68, Kruskal-Wallis *H*-test). Even cases in which preoperative head collapse had progressed by 3 mm or more, a favorable trend of necrotic lesion repair was observed (Fig. 3c).

## DISCUSSION

Hip arthroplasty performed for the femoral head osteonecrosis of young patients who were highly active had a high-failure rate; thus joint preservation treatment should be the desired treatment option for such patients [3].

The primary principle of femoral head rotational osteotomy is to move the residual viable region to the weight-bearing area through rotation and to move the necrotic lesion away from the main weight-bearing area. Its secondary principle is to achieve joint stability by reducing the femoral head from its subluxed position to its centripetal position [25].

Sugioka reported that a mean of 11-year post-operative follow-up was performed on 295 joints for which anterior rotational osteotomy was conducted, and 86% of the cases showed good to excellent results; However, in cases with 2 mm or more progression of head collapse, results were poor [11]. Anterior rotational osteotomy was indicated for cases with one-third or more viable regions remaining posteriorly. However, there were not several cases in which necrotic lesion had extensively spread both anteriorly and posteriorly, rendering anterior rotational osteotomy out of indication.

HDPRO is a surgical technique that is effective for cases with an extensive necrotic lesion, which has spread both anteriorly and posteriorly, and has not been indicated for anterior rotation [18]. Anterior rotational osteotomy has been reported to create instability because of the collapsed necrotic lesion that has migrated anteriorly post-operatively [12]. In contrast, after posterior rotation, a broad spherical viable region will be located anteriorly and the collapsed necrotic lesion will be located medially to posteromedially. In daily activities the hip joint is mainly in the flexed position, resulting in the femoral head remaining constantly stable [16]. For these reasons, HDPRO is thought to create an environment in which the weight could be dispersed and stabilized, leading to the repair of the necrotic lesion [18]. In this study, in cases with extensive osteonecrosis as well as advanced head collapse, HDPRO was performed and the repair of necrotic lesions was subsequently observed at an early stage on MRI images. Particularly in teenager cases, repair was most prominent. This was considered to be because of their active tissue repair and regeneration capabilities. Traumatic femoral head osteonecrosis resulted in a better repair of necrotic lesion after HDPRO as compared with that of non-traumatic osteonecrosis. In this study, the early repair of necrotic lesion was observed on MRI. This confirmed Atsumi’s report in which the head regained its spherical contour as a means of head remodeling after HDPRO on plain X-rays both for traumatic and non-traumatic osteonecrosis [17].

It became clear that good post-operative repair of the necrotic lesion can be expected for young patients with extensive traumatic femoral head osteonecrosis despite extensive necrotic lesion. In this study, in cases with 3 mm or more head collapse and joint space narrowing, the repair of necrotic lesion after HDPRO was observed on MRI. The reason why necrotic lesion repair occurred even in cases with advanced collapse was probably because the femoral head stability can be achieved within the acetabulum post-operatively. In addition, the viable region of the head constantly bears weight, resulting in the reduction of pressure on the collapsed necrotic lesion. The results of HDPRO for extensive femoral head osteonecrosis are excellent [14–18], but because it is technically demanding, in reality it is hardly widely used [19].

It is generally thought that when extensive osteonecrosis occurs, collapse is observed early and that joint preservation becomes impossible. However, HDPRO is considered to be a useful operation which leads to early post-operative repair of the necrotic lesion, enabling joint preservation.

## CONFLICT OF INTEREST STATEMENT

None declared.
